# Cyclization and Docking Protocol for Cyclic Peptide–Protein Modeling Using HADDOCK2.4

**DOI:** 10.1021/acs.jctc.2c00075

**Published:** 2022-06-02

**Authors:** Vicky Charitou, Siri C. van Keulen, Alexandre M. J. J. Bonvin

**Affiliations:** Computational Structural Biology Group, Bijvoet Centre for Biomolecular Research, Science for Life, Faculty of Science—Chemistry, Utrecht University, Padualaan 8, Utrecht 3584 CH, The Netherlands

## Abstract

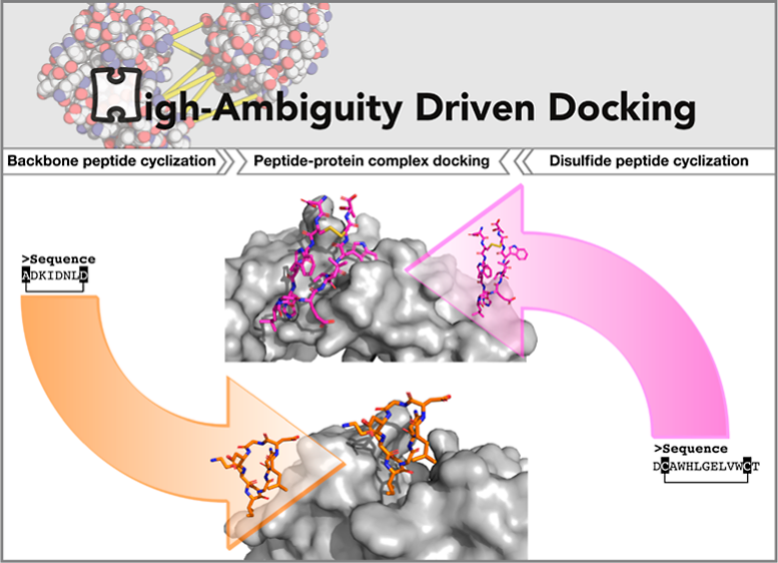

An emerging class of therapeutic molecules are cyclic peptides with over 40 cyclic peptide drugs currently in clinical use. Their mode of action is, however, not fully understood, impeding rational drug design. Computational techniques could positively impact their design, but modeling them and their interactions remains challenging due to their cyclic nature and their flexibility. This study presents a step-by-step protocol for generating cyclic peptide conformations and docking them to their protein target using HADDOCK2.4. A dataset of 30 cyclic peptide–protein complexes was used to optimize both cyclization and docking protocols. It supports peptides cyclized via an N- and C-terminus peptide bond and/or a disulfide bond. An ensemble of cyclic peptide conformations is then used in HADDOCK to dock them onto their target protein using knowledge of the binding site on the protein side to drive the modeling. The presented protocol predicts at least one acceptable model according to the critical assessment of prediction of interaction criteria for each complex of the dataset when the top 10 HADDOCK-ranked single structures are considered (100% success rate top 10) both in the bound and unbound docking scenarios. Moreover, its performance in both bound and fully unbound docking is similar to the state-of-the-art software in the field, Autodock CrankPep. The presented cyclization and docking protocol should make HADDOCK a valuable tool for rational cyclic peptide-based drug design and high-throughput screening.

## Introduction

Cyclic peptides are promising therapeutic molecules with about one new cyclic peptide drug entering the market every year.^1^ Their success lies in their favorable pharmacokinetic characteristics, such as their enhanced metabolic stability^2^ and oral availability. Cyclic peptides are usually more stable than their linear counterparts, which is mainly due to resistance to chemical or enzymatic hydrolysis.^3^ Furthermore, cyclic peptides can bind large, flat protein surfaces with high affinity and specificity,^4^ as well as disrupt protein–protein interactions.^5−8^ Cyclic peptides are therefore considered a promising compound class for therapeutic modulation of challenging protein–protein interactions.

Examples of cyclic peptides in clinical use are the immunosuppressant drug cyclosporin A,^9^ antibiotics such as vancomycin^10^ and gramicidin S,^11^ as well as antifungals.^12^ Despite their successful applications, the mode of action of the majority of these molecules is poorly understood.^13^ Currently, the optimization of cyclic peptides is mainly an empirical pursuit^14−16^ involving the synthesis of many different analogues in the hope of finding one with improved target-binding properties while often facing significant delays and synthetic challenges.^13,17−19^ New high-throughput screening approaches for macrocyclic peptides, such as RaPID,^16^ attempt to overcome these challenges. However, these methods are limited to some types of cyclic peptides and require further optimization. In these cases, computational techniques can often complement experimental work, as shown by Goldbach et al.^20^ In that study, RaPID^16^ peptide selection in combination with HADDOCK was used to predict protein–peptide complexes whose complex form was challenging to solve experimentally, demonstrating that cyclic peptide discovery could greatly benefit from computational techniques.

Similar to linear peptides, cyclic peptides present computational challenges as they include a large number of rotatable bonds, often lack secondary structural elements, and their conformational transitions might be difficult to sample due to their cyclic nature. All these aspects pose challenges for predicting protein–peptide complexes.^21,22^ To overcome such challenges, a common approach is to generate cyclic peptide conformational ensembles prior to docking. These can be obtained via methods such as Monte Carlo and molecular dynamics (MD) simulations (e.g., high-temperature MD simulations and replica-exchange MD).^23^ The generated ensemble can then be used as input to model the protein–peptide complexes. Apart from difficulties raised due to the need of generating initial cyclic conformations, these procedures can also be time-consuming and require expertise on various software packages.

To overcome this issue and offer users a single software solution, two docking packages, AutoDock^24^ and Glide,^25^ introduced a macrocycle module. Using docking instead of Monte Carlo or MD simulations for the prediction of cyclic peptide–protein complexes offers an increase in efficiency, enabling high-throughput screening of different peptides. However, peptide–protein docking remains challenging as it is difficult to incorporate the conformational sampling of such flexible peptides in docking calculations. Currently, the state-of-the-art practice for cyclic peptide docking is AutoDock CrankPep (ADCP)^26,27^ that offers a one-software pipeline to generate a cyclic peptide conformational ensemble and performs docking with the obtained cyclic peptide models. ADCP folds the peptide in the energy landscape created by the receptor, thus yielding docked peptide poses.

In this study, a new cyclic peptide cyclization and docking protocol is presented using the integrative modeling software package HADDOCK.^28^ Nine different docking protocols were benchmarked on a set of 30 cyclic peptide–protein complexes to evaluate their performance. The best-performing protocol shows that HADDOCK achieves a competitive performance in the field for both bound (holo) and unbound (apo) receptor conformations while using binding interface information on the receptor side in combination with peptide conformations generated from the sequence. More specifically, HADDOCK predicts within the top 10 HADDOCK-scored solutions of bound (holo) receptor docking at least one medium or higher-quality structure for 70% of the tested cyclic peptide–protein complexes, according to the critical assessment of prediction of interaction^29^ (CAPRI) criteria. In unbound (apo) receptor docking, HADDOCK predicts models in the same quality range within the top 10 solutions for 60% of the dataset which can be further enhanced by clustering. When only short cyclic peptides (≤10 residues) are considered, the performance of HADDOCK further increases, reaching 88.2% in bound receptor docking and 68.75% for fully unbound docking. A success rate of 100% is reached for both holo and apo receptor conditions when considering acceptable or higher-quality models for the short cyclic peptide subset. Overall, HADDOCK’s performance is comparable to or better than the current state-of-the-art practice, especially for short cyclic peptides.

## Methods

### Dataset Preparation

**Figure 1:**
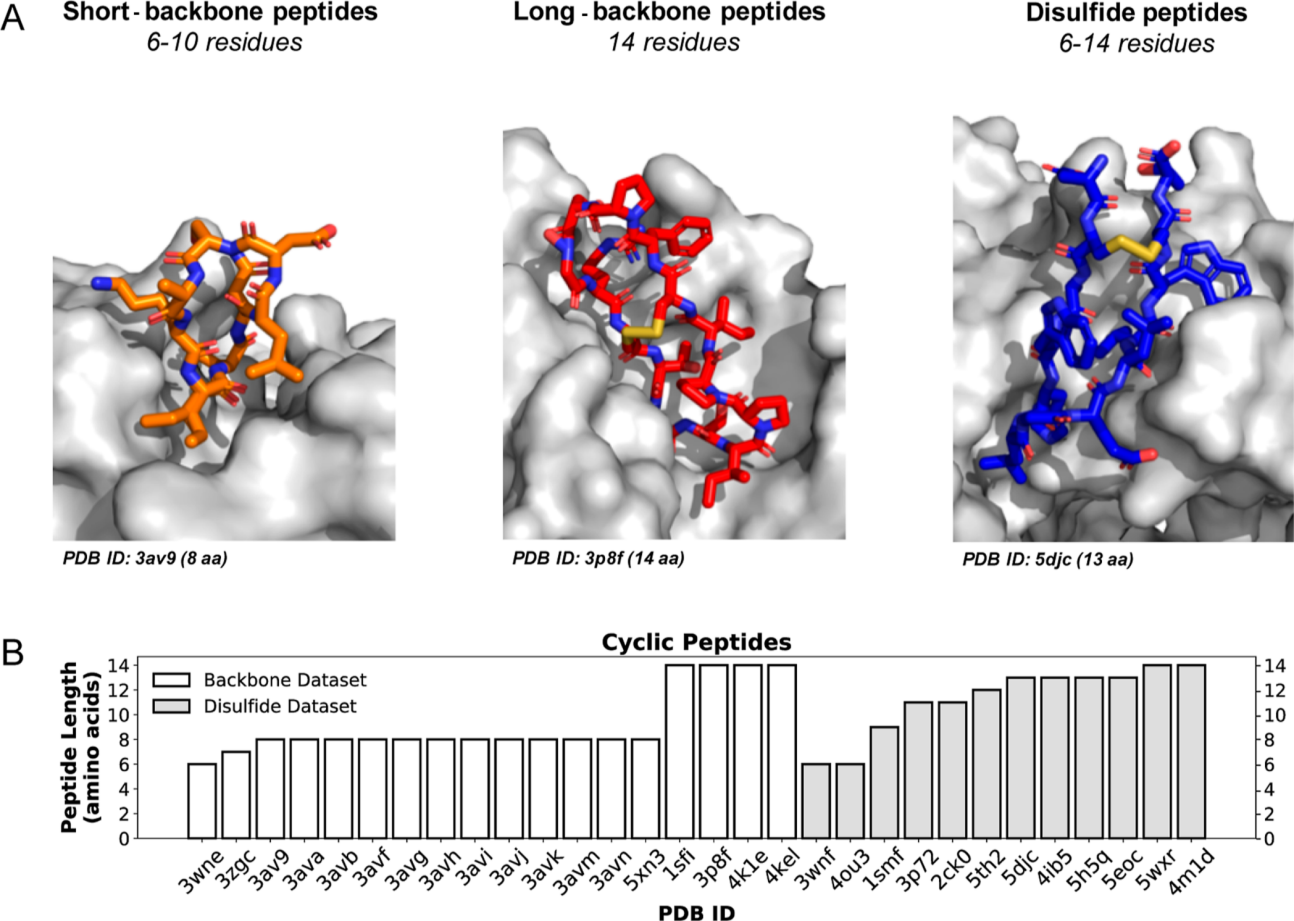
Visualization of backbone and disulfide cyclic peptide datasets. (A) Stick representation of three peptide examples from the backbone and disulfide dataset indicating their Protein Data Bank (PDB) ID and sequence length. Receptor proteins are shown in a gray surface representation and the peptides are highlighted in orange, red, and blue. Short backbone peptides (orange) consist of a maximum of 10 residues, whereas long backbone peptides (red) can include up to 14 residues and an additional disulfide bond. Disulfide peptides (blue) range in length between 6 and 14 residues. (B) Histogram of peptide sequence length for the different complexes of the backbone and disulfide datasets.

The two datasets used to benchmark the protocols in this study are composed of 30 cyclic peptide–protein complexes extracted from the dataset described by Zhang et al.^26^ The first set of complexes, the backbone dataset, includes 18 complexes in which the peptide is cyclized through its N- and C-termini with a minimum sequence length of six amino acids (Table S1). Four out of these 18 peptides include more than 10 residues and contain an additional disulfide bond (Figure 1). The second set of complexes, the disulfide dataset, includes 12 complexes which contain peptides cyclized through a single disulfide bond (Table S2).

All cyclic peptide–protein complexes in both the backbone and disulfide datasets were selected based on the following criteria: (i) length of the peptide (from 6 up to 14 residues) and (ii) formation of only one disulfide bond. In case of the disulfide dataset, an additional criterium of no more than 2 residues that would not be taking part in the cyclization was imposed as well to exclude peptides with too disordered termini (Figure 1 and Table S2). The initial conformations of the peptides were generated in PyMOL^30^ and then cyclized using distance restraints in HADDOCK (see the Peptide Cyclization Protocol).

The bound structure of each receptor in the two datasets was extracted from the corresponding PDB^31^ entry of the complex. For 25 out of 30 receptors, an unbound form (apo) was available (Tables S1 and S2). The holo protein structures were prepared for HADDOCK calculations via pdb-tools^32^ as follows:

a. First, the holo protein structures were isolated from the available complex using pdb_splitchain.
b. Then, chain ID information was removed using pdb_chain.
c. Finally, hetero atoms were removed by using pdb_delhetatm.

For the preparation of the unbound (apo) receptor structures, the same approach was followed as for the holo proteins with the exception of step (a).

In case the receptor was a multichain protein, extra steps were required to prepare the PDB file:^33^

d. Since a molecule in HADDOCK is assigned a single chain ID, no overlap in residue numbering is allowed, which means that for multichain protein, the numbering of chains might have to be shifted. For this, pdb_reres was used from pdb-tools.^32^ However, if gaps are present in the sequence of the protein, a more appropriate alternative is the pdb_shiftres script to ensure gap preservation.
e. Furthermore, in case the receptor structure contained missing loops and/or gaps, distance restraints were defined to keep the different chains together during the high-temperature flexible refinement stage of HADDOCK. These were generated with the restrain_bodies.py script from haddock-tools.^34^ The output file was saved as hbonds.tbl and was used by HADDOCK as hydrogen bond restraints by activating the hbond setting (Table S6).

### Peptide Cyclization Protocol

**Figure 2:**
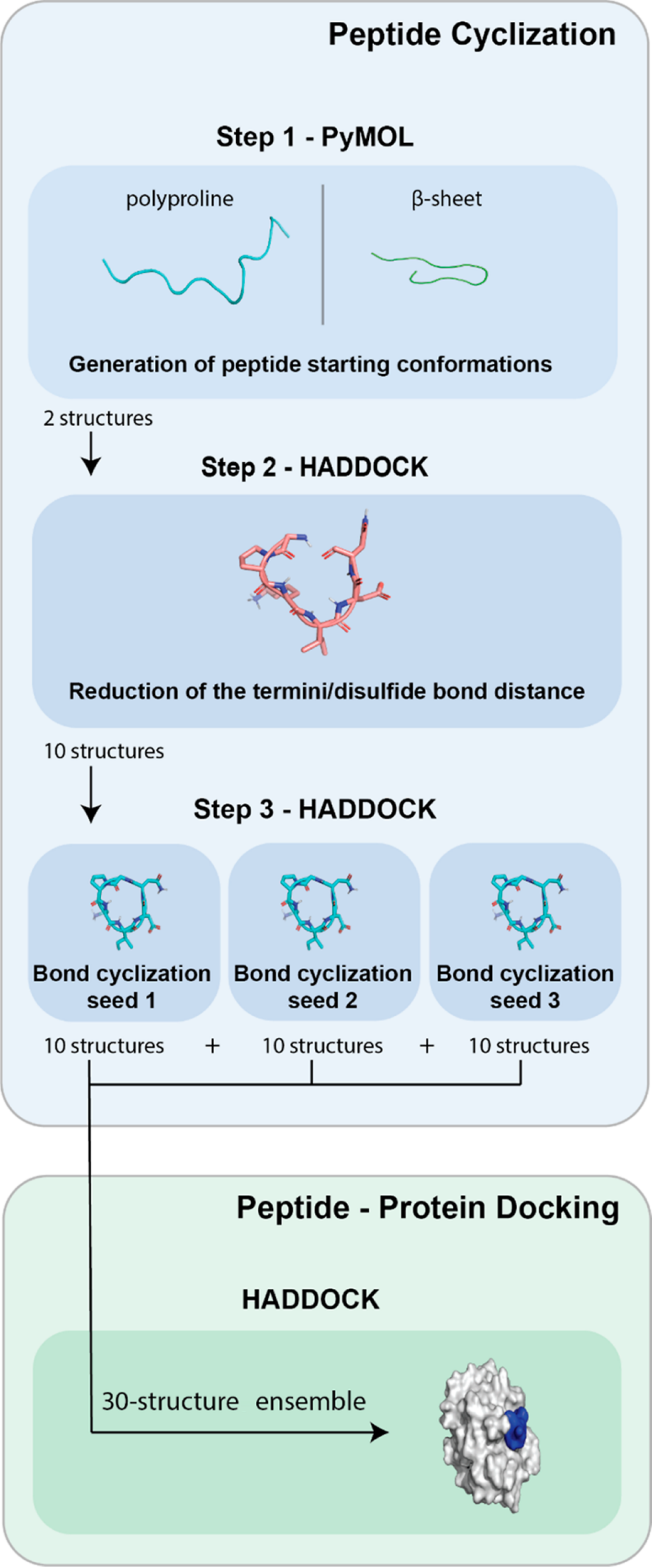
Flow chart of the peptide cyclization and docking protocol. In step 1, the starting conformations (polyproline and β-sheet) are generated from the peptide’s sequence. These two starting conformations are the input for step 2 in which the distance between the termini/disulfide bond of the peptide is reduced by applying distance restraints. 10 representative structures are used as an input for step 3. In step 3, the cyclization bond is formed, and by repeating step 3 with different seeds, the ensemble of peptide conformations is created. This ensemble is later used for docking the cyclic peptides with their respective receptors.

The cyclic peptides used for peptide–protein docking were prepared with PyMOL^30^ and the integrative modeling software package HADDOCK,^28^ version 2.4, starting from the available peptide sequences. The cyclization protocol consists of three steps (Figure 2):

1. Generating the starting conformations from the peptide sequence (PyMOL)
2. Reducing the distance between the termini/disulfide bond of the peptide (HADDOCK)
3. Cyclizing the peptide (HADDOCK)

#### Step 1: Generating the Peptide Starting Conformations

Using the PyMOL utility script build_seq, written by Robert Cambell,^35^ each peptide was generated in a beta-sheet (ss = beta) and a polyproline (ss = polypro) conformation (Figure 2). Two peptides from the disulfide dataset were crystalized with capped termini, namely, an N-terminal acetyl group (ACE) and a C-terminal N-methyl amide group (NME). For these peptides (PDB ID: 3wnf and 5h5q), a glycine residue was added to both termini, which was then mutated using the pdb-tools script pdb_mutate to an ACE or NME cap. For the other disulfide peptides, no capped N- or C- termini were resolved. Therefore, the termini of these peptides were kept positively and negatively charged by introducing an NH_3_^+^ group at the N-terminus and a negatively charged carboxylate moiety on the C-terminal side. All the other, nonterminal residues were protonated according to physiological pH levels. Subsequently, the PyMOL-generated structures were used as an input for the first cyclization step in HADDOCK, step 2.

#### Step 2: Reducing the Distance between the Termini/Disulfide Bond of the Peptide

The two starting conformations from step 1, the beta-sheet and polyproline state, were used as an ensemble of peptide conformations for the HADDOCK2.4 calculations in step 2 to generate models with a reduced distance between their termini/disulfide bond. This is a preparatory step before the actual cyclization of the peptide (introduction of the covalent bond), which is performed in step 3 (Figure 2).

The main use of HADDOCK is to perform biomolecular docking of *N* ≥ 2 molecules, but here, the software package is first used in single-molecule mode in the cyclization protocol to generate the cyclized peptides. The default HADDOCK protocol includes three stages.^28^ The first stage is rigid-body docking (it0) and is usually guided by experimental or predicted information about the protein–protein interface introduced as distance restraints. The best HADDOCK-scored models (default: 200) from it0 continue to the next stage (it1), which is a semiflexible refinement in torsion-angle space. In it1, three simulated annealing refinements are performed in which flexibility is gradually introduced into the system. After a high-temperature rigid-body search (default: 500 steps), the first cooling simulated annealing step (default: 500 steps) is performed, in which the proteins are treated as rigid bodies and their respective orientation is optimized. In the second simulated annealing step (default: 1000 steps), the side chains at the interface are allowed to move. In the third simulated annealing step (default: 1000 steps), both side chains and the backbone at the interface can move to allow for conformational rearrangements. All models from it1 move to the final refinement stage (itw) in which the protein–protein interface is refined by either an energy minimization (default in HADDOCK2.4) or by a short MD simulation in an explicit solvent.

For the cyclization of the peptides (step 2 and step 3), HADDOCK was run for a single molecule with the default docking protocol modified as follows (Tables S3 and S4):

- In it0, it1, and itw, the number of structures generated was set to 400
- In it0, it1, and itw, the cyclic peptide was defined as fully flexible
- In it0, 400 initial structures were generated while the rigid-body stage was skipped
- In it1, all steps were increased by a factor of 4
- In itw, the final refinement was performed with an explicit water shell
- The generated models were clustered using pairwise root-mean-square deviation (rmsd)^36,37^ with a cutoff of 2.5 Å.

In step 2, the electrostatic energy term is also turned off for the semiflexible simulated annealing (it1) stage (Table S3). This is because with electrostatics switched on, the generated peptide conformations tend to have their charged/hydrophilic side chains pointed toward the inside of the peptide cyclic center, leading to an unphysical conformation of the cyclic peptide, which is further away from the holo peptide state. In addition, distance restraints are introduced between the peptide’s termini/residues forming the disulfide bonds to reduce the distance between the atoms of the peptide that are involved in bond formation and induce the cyclic topology.

**Figure 3:**
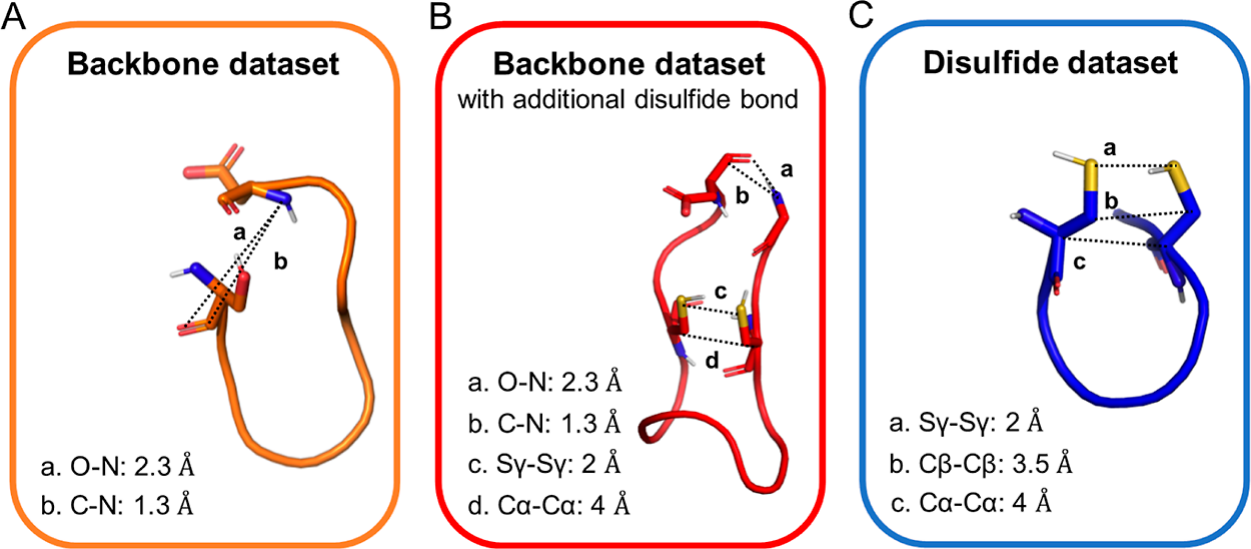
Visualization of distance restraints defined in the cyclization protocol. (A) Backbone restraints for short peptides (≤10 residues). (B) Backbone restraints for long backbone peptides including an example of an additional disulfide bond. (C) Disulfide restraints for peptides cyclized through a disulfide bond only.

- Disulfide dataset: A 4 Å distance restraint was defined between the Cα–Cα atoms of the cysteine residues that form the disulfide bond. Likewise, the distance between the Cβ–Cβ atoms was defined as 3.5 Å and the distance between the two sulfur atoms was defined as 2 Å. These values (Cα–Cα: 4 Å, Cβ–Cβ: 3.5 Å, and Sγ–Sγ: 2 Å) correspond to the measured mean distances in such cyclic peptides.^38,39^ For all distance restraints, upper- and lower-bound corrections were set to 0.1 Å (Figure 3A).
- Backbone dataset: To introduce the cyclic peptide, bond distance restraints were defined between the C and N atoms (1.3 Å) and O and N (2.3 Å) with an upper- and lower-bound correction of 0.1 Å (Figure 3B). These values represent the mean distance between the respective atoms in a peptide bond.^40^ The O–N distance is defined to restrain the O–C–N angle. When peptides are cyclized through their termini and include an additional disulfide bond (PDB ID: 1smf, 4k1e, 4kel, and 3p8f), all aforementioned disulfide and backbone distance restraints (termini [C–N: 1.3 Å, O–N: 2.3 Å] and disulfide [Cα–Cα: 4 Å, Sγ–Sγ: 2 Å]) were used except for the Cβ–Cβ restraint, which proved not to be required in this case (Figure 3C).

#### Step 3: Forming the Covalent, Cyclic Bond of the Peptide

From the modeled peptides of step 2, one representative model was selected from each of the 10 most populated clusters after rmsd clustering (Figure 2). The center of each selected cluster was used as a representative model. These 10 representative models composed the ensemble of peptide conformations used in step 3 of the cyclization protocol.

Similar to step 2, the electrostatic energy term was turned off in it0 and it1 during cyclization in step 3. The same unambiguous distance restraints used in step 2 for the termini/disulfide bonds were included in step 3 as well. In addition, the cyclic peptide option in HADDOCK is set to true for the covalent bond to be defined [provided the N and C atoms are within 3.5 Å distance (increased from the default 2 Å)]. The disulfide bond is automatically recognized and defined, provided the S–S distance is ≤4 Å (increased from the default 3 Å) (Table S5).

The difference in step 3 compared to step 2 is that the restraints are only implemented in the first two stages of docking (it0 and it1). The final stage, itw, is an extended water refinement with distance restraints switched off and water steps increased by a factor of 2 (Table S4). Hence, the final itw stage is an unrestrained very short MD refinement of the peptide models.

#### Generating the Ensemble of Cyclic Peptide Conformations with Step 3 for Peptide–Protein Docking

From the third and final step in the peptide cyclization protocol, a maximum of 10 representative models are selected out of the 10 most populated clusters after rmsd clustering. If less than 10 clusters are formed during step 3, only the representative structures from the available clusters are used. The selected models correspond to the center of the cluster—called representative models in the following. The ensemble of cyclic peptide conformations obtained from step 3 is subsequently used for peptide–protein docking with the bound (holo) or unbound (apo) form of the receptor in HADDOCK (see below). The size of the cyclic peptide ensemble can be increased by repeating step 3 with a different initial HADDOCK seed, which is used by the CNS random number generator to produce different initial velocities for the peptide. For example, by repeating step 3 three times with a different seed, a peptide ensemble with a maximum of 30 conformations can be generated for each peptide (Figure 2). By increasing the number of cyclic peptides in the ensemble, a larger variation in peptide conformation can be included during docking.

### Cyclic Peptide–Protein Docking Protocol

After the peptide cyclization process, the obtained cyclic peptide ensemble and the respective receptor structure are used to perform peptide–protein docking with HADDOCK2.4. Nine different docking protocols were tested in order to find the one leading to the best quality of docked models (Table 1). These protocols differ in the size of the cyclic peptide ensemble, the flexibility treatment, and the presence/absence of a final refinement in an explicit solvent in itw. The docking protocols were benchmarked on the backbone dataset using the holo (bound) structure of their receptor. The backbone dataset was chosen for this as its size is larger than the disulfide peptide dataset. Protocol benchmarking was done using the holo receptor rather than the apo structure to concentrate on the peptide part of the docking setup and to evaluate the effect of the various settings.

**Table 1:** Overview of the Tested Peptide–Protein Docking Protocols

protocol	cyclic peptide ensemble^a^	extra flexibility^b^	explicit solvent shell^c^	ID
1	5	OFF	OFF	5STR
2	30	OFF	OFF	30STR
3	40	OFF	OFF	40STR
4	50	OFF	OFF	50STR
5	60	OFF	OFF	60STR
6	50	ON	OFF	50STR_FLEX
7	50	OFF	ON	50STR_SOLVSHELL
8	50	ON	ON	50STR_FLEX_SOLVSHELL
9	50	ON/OFF^d^	ON/OFF^d^	50STR_COMB

a Maximum number of conformations in the cyclic peptide ensemble.

b Parameters changed in run.cns for peptide flexibility when the peptide is defined as molecule 2: nfle_2 (changed from 0 to 1), start_fle_2_1, and end_fle_2_1 (changed to the residue numbering of the first and last residue, respectively).

c Parameters changed in run.cns file for the itw stage: solvshell (changed from false to true).

d Extra flexibility and explicit solvent is switched on for long cyclic peptides (>10 residues) and switched off for short cyclic peptides (≤10 residues).

All nine protocols include the same definition of the interface on the receptor side, which is used by HADDOCK to guide the docking process. Ambiguous interaction restraints (AIRs) describing the interaction region were used to guide the docking in which the peptide was defined as fully passive and the receptor-binding region as active. Active residues are usually defined as the solvent-accessible residues experimentally identified to be involved in the interaction, whereas passive ones are the solvent-accessible surface neighbors of active residues not necessarily involved in the interaction. These passive residues are not penalized if not at the interface in the final model, while active residues not at the interface will generate an energetic penalty. Here, we defined the whole peptide as passive since it is solvent-accessible, but not all of its residues are necessarily involved in the interaction, and the receptor’s binding site as active (i.e., we assume knowledge of the binding site on the receptor).^41^ The protein interface was defined by taking into account all receptor residues within a 5 Å distance cutoff of the cyclic peptide in the available holo complex structure. By defining the peptide as fully passive, regions of the peptide that do not contact the protein will not be penalized.^41^ This AIR definition is in line with the best practice guide for peptide–protein docking with HADDOCK.^42^ The AIR file that was used during the docking calculations was generated by using the active-passive-to-ambig script from haddock-tools.^34^

The base docking setup in all nine protocols includes the following parameter changes with respect to the default settings (Table S6):

- The peptide was defined as cyclic by activating the cyclic peptide option in run.cns.
- In it0, it1, and itw, the number of models docked were increased to 5000, 400, and 400, respectively.
- In it1, all steps in the semiflexible simulated annealing stage were increased by a factor of 4 to allow for more conformational sampling of the peptide in the context of the receptor.
- Generated models were clustered based on the interface ligand rmsd using a 5 Å cutoff.

#### Assessing the Impact of the Peptide Ensemble Size (Protocols 1–5)

In the first five protocols, the maximum number of conformations in the cyclic peptide ensemble were varied from 5 to 60. Protocol 1 includes the smallest ensemble of peptide conformations of five representatives selected from the top five most populated clusters, generated using a single seed. In protocols 2–5, the size of the ensemble was increased by using 3, 4, 5, or 6 different seeds and selecting a maximum number of 10 representatives from each seed, depending on the number of available clusters. The total number of representatives used per protocol is shown in Tables S7 and S8.

#### Assessing the Impact of Allowing for Extra Flexibility and Use of an Explicit Solvent Shell (Protocols 6–9)

In protocols 6–9, the effect of extra flexibility and explicit solvent refinement was assessed. Because of the intrinsically high flexibility of peptides, the optimal setting for protein–peptide docking of linear peptides is to define the peptides as fully flexible and to refine them in an explicit solvent shell. By defining a peptide as fully flexible, both the side chains and the backbone of the entire peptide are allowed to move throughout the docking, except for the initial rigid-body minimization stage (it0). To test whether this setting for linear peptides is also required for cyclic ones, the peptides were defined as fully flexible throughout stage it1 and itw in protocol 6. On the other hand, to evaluate the impact of explicit solvent-shell refinement in cyclic peptides, protocol 7 was used, in which the peptides were refined in explicit solvent (water), but the default flexibility was maintained. Moreover, to test the combined effect of these two settings in protocol 8, peptides were both defined as fully flexible and refined in an explicit solvent shell. Finally, in protocol 9, peptides were treated differently based on the length of their sequence. For peptides that are composed of 10 residues or less, the default flexibility was applied and they were only subjected to a final energy minimization. Peptides longer than 10 residues were treated as fully flexible throughout it1 and itw and refined in an explicit solvent shell.

### Success-Rate Analysis Based on the Fraction of Native Contacts

The quality of the generated models was assessed using the fraction of native contacts (*f*_nat_). This metric is defined as the number of native (true) residue–residue contacts in the predicted complex at the protein–peptide interface, divided by the number of contacts in the reference crystal structure of the complex.^29^ A pair of residues on different sides of the protein–protein interface was considered to be in contact if any of their heavy atoms are within a 5 Å distance cutoff. According to the CAPRI^43^ criteria for protein–peptide complexes, models with an *f*_nat_ above 0.2 were ranked as acceptable, above 0.5 as medium, and above 0.8 as high quality. The interface rmsd (i-rmsd) is another metric typically used for interface accuracy. However, the i-rmsd is less well suited for cyclic peptide analysis, especially for cases in which flexible terminal extensions exist outside the cyclic structure (i.e., for the disulfide dataset),^26^ which is discussed in the Supporting Information Methods Section and Figure S1. Nevertheless, i-rmsd values were also calculated for the generated models along with *f*_nat_ and are reported in the Supporting Information (see Table S11). According to the CAPRI^43^ criteria for protein–peptide complexes, models with i-rmsd values below 2.0 were ranked as acceptable, below 1.0 as medium, and below 0.5 as high quality.

The performance of each tested protocol was evaluated using the *f*_nat_ success rate. The success rate for single-structure analysis is defined as the percentage of cases in which at least one model of a given accuracy (high, medium, or acceptable) is found within the top *N* solutions ranked by HADDOCK (*N* = 1, 5, 10, 20, 50, 100, 200) using the itw scoring function.^44^ For example, a success rate of 60% for medium-quality structures in a top 10 means that in the top 10 HADDOCK-ranked solutions at least one model of medium or higher quality was found for 60% of the complexes of the dataset. Regarding the cluster analysis, the success rate is calculated for the four best clusters, according to the itw HADDOCK score, using the top four structures per cluster. The cluster success rate is defined as the percentage of cases in which at least one model of a given accuracy (high, medium, or acceptable) within the top four members of the cluster is found within the best *N* clusters ranked by the itw HADDOCK score (*N* = 1, 2, 3, 4).

## Results and Discussion

As the quality of the generated cyclic peptide structures can greatly influence the peptide–protein docking performance, the quality of the cyclic peptide ensembles generated with HADDOCK is first discussed. Subsequently, the different docking protocols are compared to determine the optimal settings for cyclic peptide docking. These have been tested using the backbone dataset and the holo structure of the receptor. The best cyclic peptide docking protocol was then applied to fully unbound docking using the apo receptor structure for both the backbone and disulfide datasets. Next, the optimized protocol’s performance is compared to the baseline docking performance for both datasets using the holo receptor and single holo-cyclic peptide conformation. Finally, the HADDOCK docking results are compared to the results of cyclic peptide docking using ADCP.

### Quality Assessment of the Cyclic Peptide Conformational Ensemble

Prior to docking, the quality of the generated cyclic peptide ensembles was analyzed by calculating their backbone rmsd’s with respect to the holo-cyclic peptide conformation.

#### Peptide Ensemble of the Backbone Dataset Is Closer to the Holo State than the Disulfide Dataset

**Figure 4:**
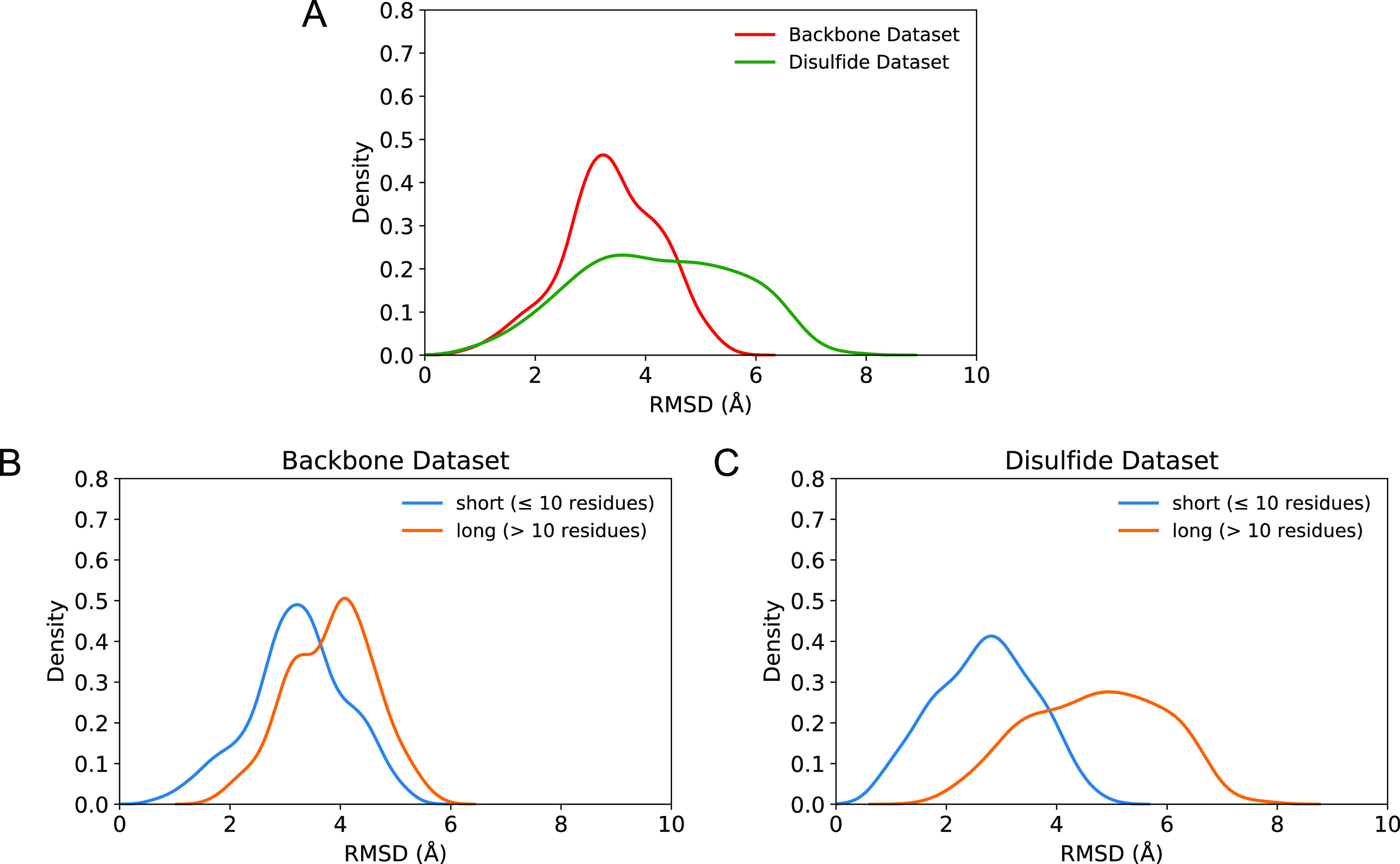
rmsd distribution of the 50-structure cyclic peptide ensemble. (A) Backbone rmsd distribution of the generated backbone peptides, depicted in red, calculated by using 835 data points (18 peptides*representative structures/seed), while 416 data points were included for the disulfide dataset shown in green (12 peptides*representative structures/seed). The number of representative structures per seed varies between 1 and 10 for every peptide and depends on the number of clusters formed at the end of the cyclization step (step 3) (Tables S7 and S9). For PDB entries 2ck0, 4ou3, 4ib5, 5h5q, 5djc, and 5wxr of the disulfide dataset, the residues outside their cyclic region were excluded during the rmsd calculation. (B) Backbone rmsd distribution of the short (blue) and long (orange) backbone peptides was calculated using 635 (14 peptides*representative structures/seed) and 200 (4 peptides*representative structures/seed) data points, respectively (Table S7). (C) The backbone rmsd distribution of the short (blue) and long (orange) disulfide peptides was calculated using 98 (3 peptides*representative structures/seed) and 318 (9 peptides*representative structures/seed) data points, respectively (Table S8). For PDB entries 2ck0, 4ou3, 4ib5, 5h5q, 5djc, and 5wxr of the disulfide dataset, the residues outside their cyclic region were excluded during the rmsd calculation.

When considering the best RMSD’s obtained within the tested ensemble sizes (from 5 to a maximum of 60 structures), the average rmsd value of the best structures becomes lower by enlarging the ensemble (Tables S9 and S10). This trend suggests that by enlarging the peptide conformational ensemble, the probability of including a peptide structure resembling the holo peptide conformation is increased. The presence of a near-holo peptide conformation in a peptide ensemble could potentially lead to enhanced docking results. The rmsd analysis of the 50-structure peptide ensemble shows that the modeled peptides from the backbone dataset are closer to their holo crystal structure than the cyclic disulfide ones (Figure 4A). On average, the best-generated backbone peptides of each ensemble have an rmsd of 1.5–2 Å, while the best disulfide peptides only reach an rmsd of 3 Å (Tables S9 and S10).

A reason for the difference in ensemble quality between the two datasets could be the flexible termini of the disulfide peptides. However, in the rmsd calculations of the disulfide dataset, only the cyclic regions of the peptides were considered to eliminate differences caused by disordered termini. Therefore, the impact of peptide length was assessed next as extending the peptide length increases the number of degrees of freedom, leading potentially to a higher backbone rmsd.

#### Conformational Quality of the Short Cyclic Peptides Is Higher than the Quality of the Long Peptides in Both Datasets

Regarding the quality of the generated cyclic peptides separated by sequence length, the shorter peptides (≤10 residues) are closer to their holo crystal structure (Figure 4B,C) than the long cyclic peptides. The backbone dataset consists of mainly short (14 out of 18) peptides (Table S1), which could explain the overall higher quality of the conformational ensemble compared to the disulfide peptides which only contains 3 short peptides out of 12 (Figure 4A) (Table S2). This difference in structural quality between short and long peptides can be observed in both the backbone and the disulfide datasets and points to the challenges imposed by ab initio modeling when the number of rotatable bonds increase.^45^

### Cyclic Peptide–Protein Docking Protocol Optimization

After the quality assessment of the generated peptide ensembles, optimization of the cyclic peptide–protein docking protocol was performed. Nine protocols were tested to determine the best settings for cyclic peptide docking using HADDOCK2.4. The performance of the different protocols was assessed by using *f*_nat_ as a metric for the success-rate analysis (see Methods). Both single-structure and cluster analyses were performed for the best-performing protocol (Figure S2).

The inherent flexible nature of peptides poses difficulties in docking due to the possible conformational changes occurring upon binding. Since cyclic peptides are a subcategory of peptides, the search for the optimal docking settings was based on the previously proposed HADDOCK protocol for linear peptide–protein docking,^46,47^ a protocol that attempts to overcome the aforementioned difficulties by including the following features:

- An ensemble of three peptide conformations (beta, polyproline-II, and alpha helical) is used for docking.
- In it1 and itw, the peptides are defined as fully flexible.
- In itw, the models are refined via a short MD simulation in an explicit solvent shell (the default in HADDOCK2.2).

For cyclic peptide–protein docking, the effect of these three settings was evaluated by performing the protocols described in Table 1. First, the size of the peptide conformational ensemble was assessed by comparing the results of protocols 1–5 (Table 1) in which the peptide ensemble size increases from 5 to 60 conformations. Second, protocols 6–9 (Table 1) were conducted to determine the effect of extra flexibility and explicit solvent-shell refinement.

#### 50 Peptide Structures Is the Optimal Ensemble Size for Cyclic Peptide Docking

**Figure 5:**
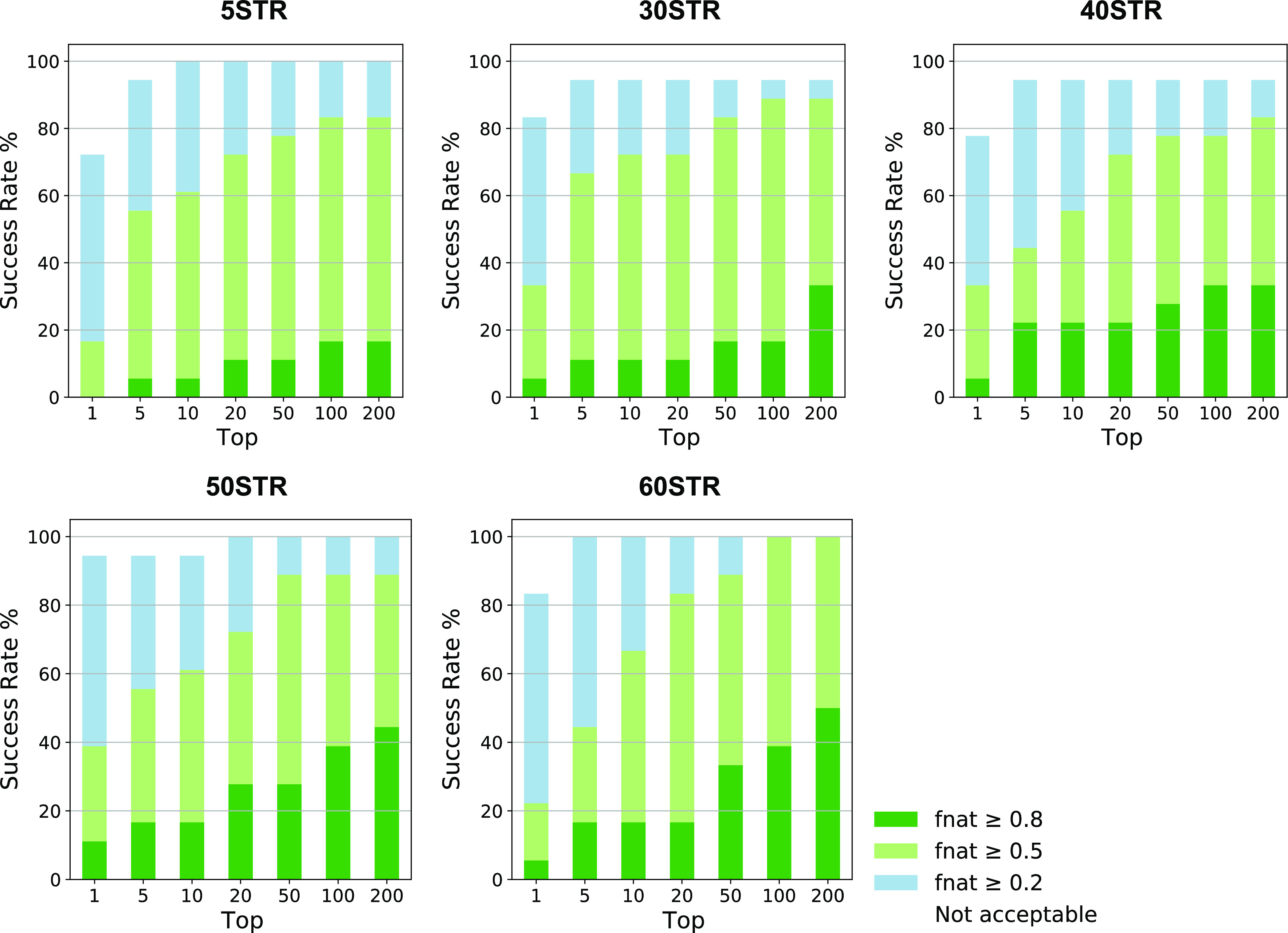
*F*_nat_ success-rate plots for 5STR, 30STR, 40STR, 50STR, and 60STR docking protocols. Plotted are the success rates (%) of protocols 1–5, including a 5-, 30-, 40-, 50-, or 60-structure ensemble. The color coding (blue, light green, and green) indicates the quality of the models, from acceptable to medium and high quality, according to the CAPRI criteria.

To identify the optimal number of structures per ensemble, experiments of various ensemble sizes were performed. Ensembles of 5, 30, 40, 50, and 60 structures (5STR, 30STR, 40STR, 50STR, and 60STR, respectively) were generated per peptide included in the backbone dataset (see Methods). In order to concentrate on the peptide conformational sampling, the docking was performed with their respective holo receptor structure (Tables 1 and S5). The results in Figure 5 show that increasing the number of structures of the ensemble from 5 to 30 leads to an increase of 11.1% in the success rate of medium or high-quality structures within the top 10 HADDOCK-scored solutions. A further expansion of the ensemble to 50 structures resulted in complex models that continued showing this ascending trend in performance. The medium or higher-quality success rates with a 50-structures ensemble were 55.5% for the top 5 HADDOCK-ranked solutions and 61.1% for the top 10 (Figure 5).

However, for the success rates of the 60-structure ensemble experiment, a reduction in quality can be observed in comparison to the 50-structure setup (Figure 5). A reason for this reduction could be the “dilution effect”, which is caused by enlarging the peptide ensemble without increasing the number of models generated in the initial rigid-body docking (it0, 5000), leading to a decrease in sampling per conformation during docking. However, after increasing the number of generated it0 models to 10,000 in the 60-structure setup, no significant increase in performance was observed (Figure S3). Another reason for the reduction in model quality could be the scoring of the generated models: by adding more conformations to the ensemble, challenges could be introduced to the scoring of the docked models, leading to more false-positive solutions to be ranked higher in the top. This is illustrated in Figure 5: although the 60STR experiment generates more medium- and high-quality structures in the top 200, the scoring function fails to rank them within the top 10. Reoptimization of the scoring function for cyclic peptides could potentially improve the scoring.

#### Extra Flexibility and Explicit Solvent Refinement Are Required Only for Long Peptides (>10 Residues)

After identifying the best-performing ensemble size, the effect of extra flexibility and explicit solvent-shell refinement during cyclic peptide docking was evaluated. In our previous work on linear peptide structures, the peptides were treated as fully flexible and refined in an explicit solvent shell.^47^ To evaluate if these settings are also optimal for cyclic peptide–protein docking, the 50STR_FLEX protocol was performed, in which peptides are defined as fully flexible during the it1 and itw stages of docking. Besides 50STR_FLEX, the 50STR_SOLVSHELL setup was tested in which complexes undergo explicit solvent-shell refinement via a short MD simulation in water. Finally, the 50STR_FLEX_SOLVSHELL protocol was evaluated, including both full flexibility and explicit solvent refinement. The results were compared with the success rates of the 50STR protocol, in which both extra flexibility and explicit solvent-shell refinement were turned off (Table 1).

**Figure 6:**
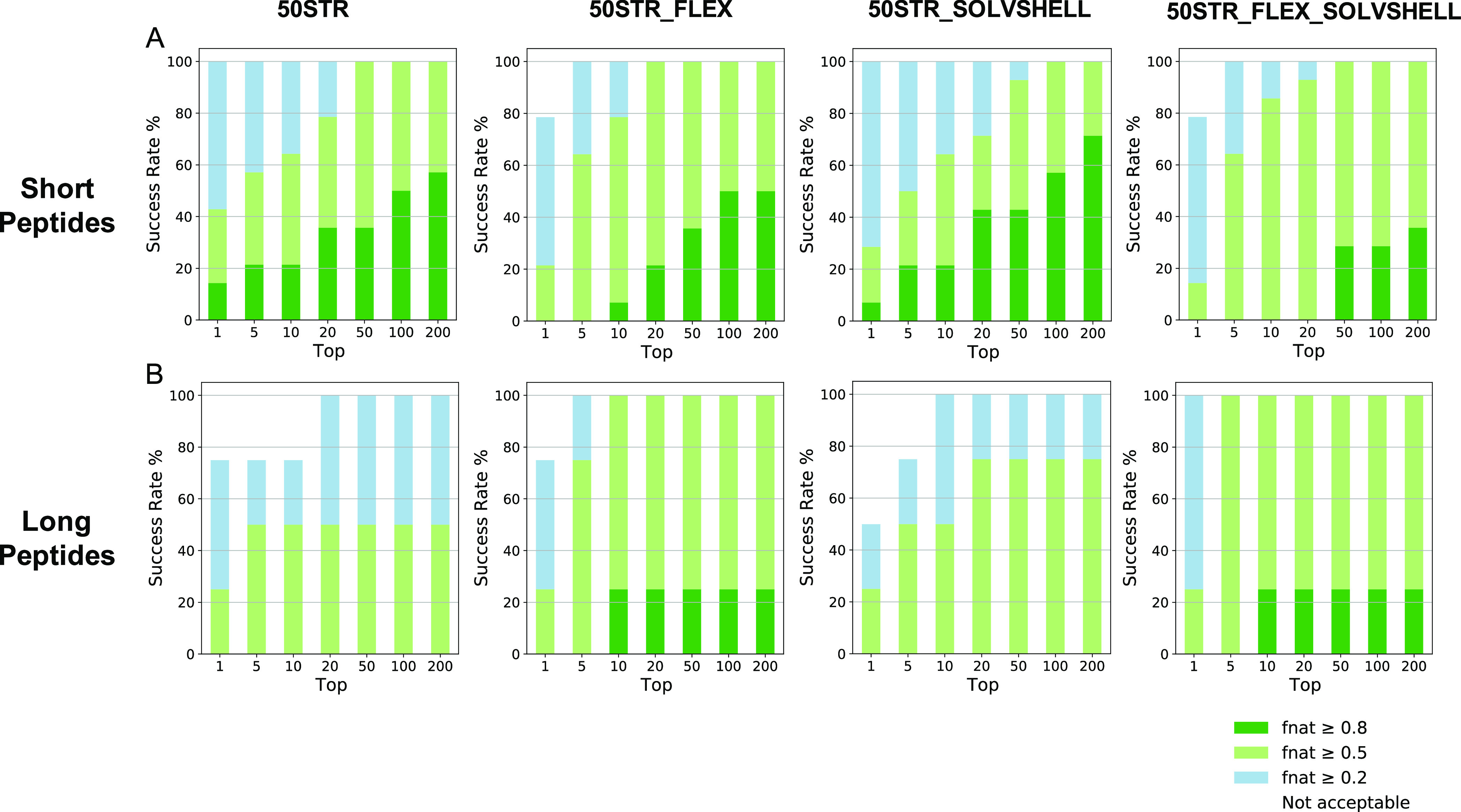
*F*_nat_ success-rate plots of 50STR, 50STR_SOLVSHELL, and 50STR_FLEX docking protocols. (A) Plotted are the success rates for the short peptides (14 out of 18 complexes), ≤10 residues, of the backbone dataset for four experiments (50STR, 50STR_FLEX, 50STR_SOLVSHELL, and 50STR_FLEX_ SOLVSHELL). The color code (from blue to green) indicates model quality (from acceptable to high, respectively) according to the CAPRI criteria. (B) Plotted are the success rates of the long peptides (4 out of 18 complexes), >10 residues, of the dataset for four tested protocols (50STR, 50STR_FLEX, 50STR_SOLVSHELL, and 50STR_FLEX_ SOLVSHELL). The color code (from blue to green) indicates model quality (from acceptable to high, respectively) according to the CAPRI criteria.

When considering the complete backbone dataset, no clear answers are obtained regarding the impact of extra flexibility and explicit solvent-shell refinement on the quality of the generated models (Figure S4). However, as shown in Figure S5, increased flexibility does appear to generate more acceptable- or higher-quality models for the long peptides. Therefore, short and long peptides were analyzed separately to investigate the effect of peptide length on the prediction performance (Figure 6).

For short cyclic peptides (≤10 residues), adding extra flexibility and/or explicit solvent refinement has no or a slightly negative impact on the docking performance (Figure 6A), whereas these two features do improve the prediction quality for long cyclic peptides (>10 residues) (Figure 6B), with the extra flexibility having the most impact. These results suggest cyclic peptides should be treated differently during docking according to their length. Long cyclic peptides (>10 residues) show more similarities to linear peptides than short cyclic peptides and benefit from including extra flexibility and an explicit solvent shell. On the other hand, the optimal performance for short cyclic peptides (≤10 residues) is achieved by using the default flexibility setting and the default final energy minimization in itw (HADDOCK 2.4).

#### Optimized Docking Protocol Performs Better for the Backbone than for the Disulfide Dataset

**Figure 7:**
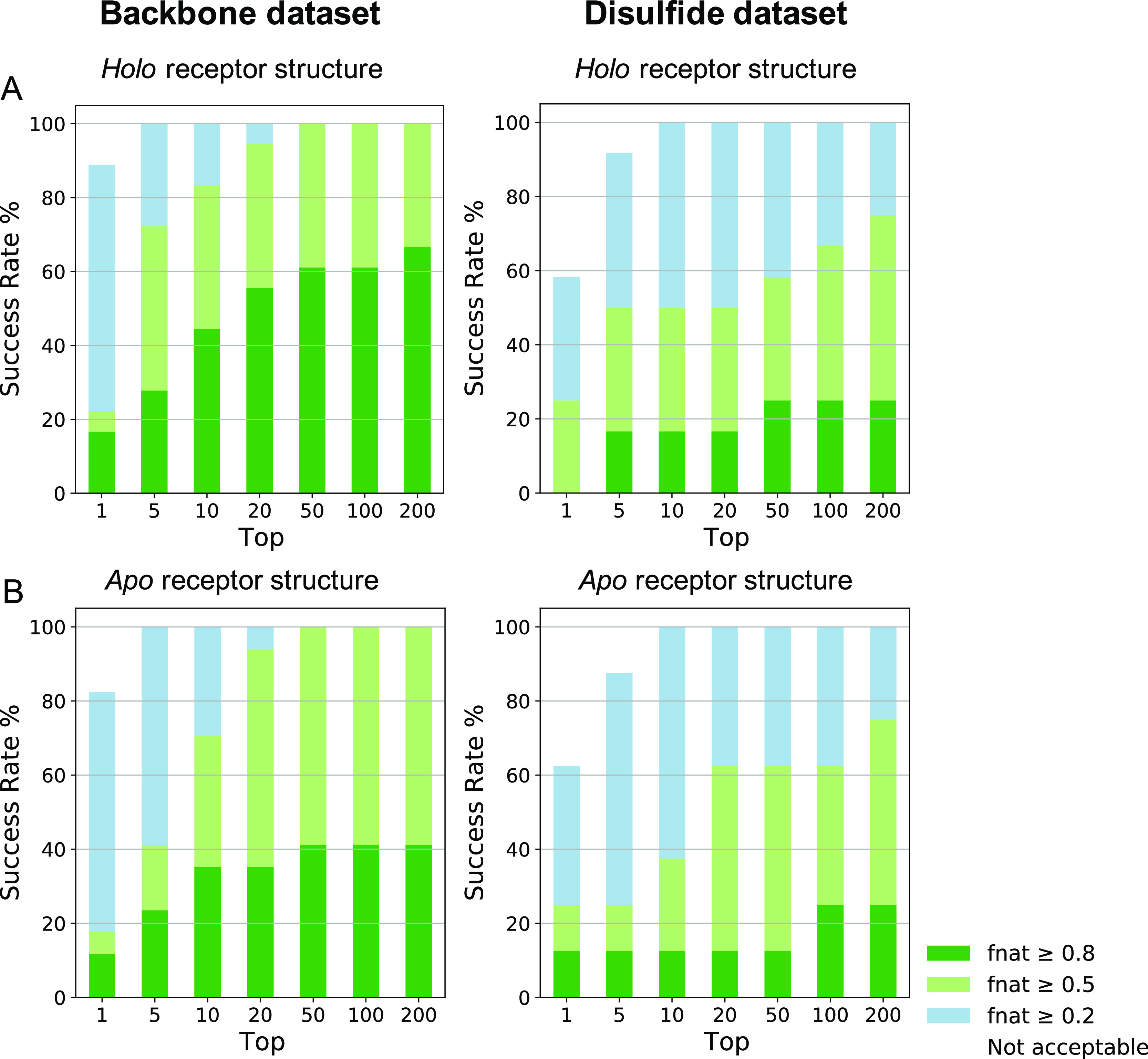
*F*_nat_ success-rate plots for 50STR_COMB docking protocol in both datasets. (A) Plotted are the docking success rates using the holo receptor for the backbone (18 complexes) and the disulfide dataset (12 complexes). Color coding (from blue to dark green) indicates model quality (from acceptable to high) according to the CAPRI criteria. (B) Plotted are the docking success rates using the apo receptor for the backbone (17 complexes) and the disulfide dataset (8 complexes). Color coding (from blue to dark green) indicates model quality (from acceptable to high) according to the CAPRI criteria.

After characterization of the optimal ensemble size and the effect of flexibility and solvent refinement on cyclic peptide docking, the best-performing protocol was identified to be 50STR_COMB. This protocol uses a maximum of 50 structures in the peptide ensemble that is used as input for docking and treats peptides differently during the docking protocol, depending on their sequence length. For short peptides, ≤10 residues, the 50STR protocol is applied, while the long peptides, >10 residues, are docked using the 50STR_FLEX_ SOLVSHELL setup, allowing for extra peptide flexibility. When using this optimized docking protocol for the holo receptor, HADDOCK could generate acceptable or higher-quality solutions for every complex of the dataset within the top 10 HADDOCK-scored structures (Figures S6 and S7). When individually assessing the backbone and disulfide dataset, HADDOCK shows success rates of 83.3 and 50% for medium- or higher-quality structures within the top 10 models (holo receptor setup), respectively (Figure 7A).

The largest impact of introducing structural flexibility (it1 and itw) is on the model quality measured by *f*_nat_: from the it0 to the it1 stage, an *f*_nat_ improvement of almost 0.25 on average is obtained, while the effect of itw is less pronounced (0.05 on average) (Figure S7). This finding is in line with our previous work on peptide–protein docking,^47^ showing that flexibility of the system is mainly handled during the flexible refinement stage (it1) of HADDOCK, while the water refinement stage (itw) has the most impact on the energetics.

To determine the performance of HADDOCK in the more realistic apo setup (fully unbound), the optimal docking protocol (50STR_COMB) was performed using the apo form of the receptor and the generated peptide ensembles for both datasets. For the backbone dataset, a success rate of 70.6% medium or higher quality in the top 10 solutions (Figure 7B) was obtained, while this was 37.5% for the disulfide dataset (Figure 7B). Note that when considering acceptable or higher solutions, both holo and apo receptor docking lead to a 100% success rate in the top 10 for both datasets and even 100% in the top five for the backbone set.

The decrease in docking success between the backbone and the disulfide dataset mainly seems to be related to differences in the quality of the starting peptide conformational ensembles. As shown previously, the rmsd analysis of the peptide conformational ensembles revealed that the backbone-generated peptides were of better conformational quality than the disulfide peptides due to a larger number of long peptides included in the disulfide dataset with respect to the backbone peptides (Figure 4). The docking performance difference between the two datasets appears to be related to the short/long peptide ratio of each dataset (Figures S5, S6, and S8).

Analysis of the 50STR_COMB generated models showed that reoptimization of HADDOCK’s scoring function for cyclic peptides could be required to rank all high-to-medium-quality models within the top 10. High-quality structures are predicted, but these are often ranked among the top 100 solutions (Table S11), making it impossible for a user to extract them. Clustering of the models according to their interface ligand rmsd can partially compensate for the scoring challenges imposed as the model dimension is significantly reduced, leaving only representative models of each cluster to be considered by the user (Figure S2). These include the top four HADDOCK-scored models per cluster, which are used to calculate the overall cluster score (16 models for the best 4 clusters).

### Comparison with a Best-Case Scenario (Bound Docking)

The 50STR_COMB protocol has been determined as the optimal docking protocol for cyclic peptides, and its performance in both the backbone and the disulfide dataset has been assessed. However, it is worthwhile comparing the success rates of the 50STR_COMB protocol with the success rates of the best-case (but unrealistic) scenario in which the holo cyclic peptide conformation is used instead of a modeled 50-structure peptide ensemble to dock with the holo receptor. In this best-case scenario, no conformational changes are required for an optimal interaction between peptide and receptor. Since the input of these bound docking experiments uses the holo X-ray structures, their success rates portray the optimal performance of HADDOCK given the knowledge of the binding site on the receptor and the absence of conformational changes (Figure S9).

The best-case scenario reaches a 94.4% success rate for medium- or higher-quality models in the top 10 for the backbone dataset and 66.7% for the disulfide dataset (Figure S9). When only considering the top one (best ranked) HADDOCK model, the success rate remains quite high with 83.3 and 41.6% for the backbone and the disulfide dataset, respectively. If only the short peptides are taken into account, the success rate for medium-to-high-quality models in the top 10 becomes 92.9% for the backbone dataset and 100% for the disulfide dataset (Figure S10). This indicates that long peptides cyclized through a disulfide bridge (>10 residues) are not as well predicted in the best-case scenario compared to short peptides. This difference in peptide performance with the 50STR_COMB protocol (Tables 1, S6, and S8) could be due to the fully flexible definition for all long peptides and refinement in an explicit solvent. The conformations can thus move away from their bound form during flexible refinement. On the other hand, only the default flexibility and energy minimization in itw are applied to the short peptides (≤10 residues), resulting in a reduction in conformational sampling for short peptides with respect to the long ones.

These results indicate that HADDOCK is a powerful tool for short cyclic peptide–protein complex structure prediction when the peptide’s conformation is known (which could be the case, e.g., when NMR data allow to define the conformation of the bound peptide). As discussed previously, unbound docking resulted in a medium-to-high-quality success rate of 83.3% for the backbone and 50% for the disulfide dataset in the top 10 (Figure 7). The comparison of the best-case scenario results with the unbound docking results demonstrates that the quality of the initial peptide structure(s) greatly determines the quality of the achievable docking results.

### Comparison with the State of the Art

#### HADDOCK’s Cyclic Peptide–Protein Docking Performance Is Comparable to That of ADCP

In Figure S11, the same dataset is used to compare the complex predictions of ADCP^26^ with those obtained using our optimized protocol (50STR_COMB). The overall HADDOCK success rate, including both backbone and disulfide datasets, is 70% for medium-quality structures or higher (top 10) when docked to the holo receptor, which is equal to the ADCP success rate. This drops to 60% when docked to the apo receptor against 76% for ADCP.

**Figure 8:**
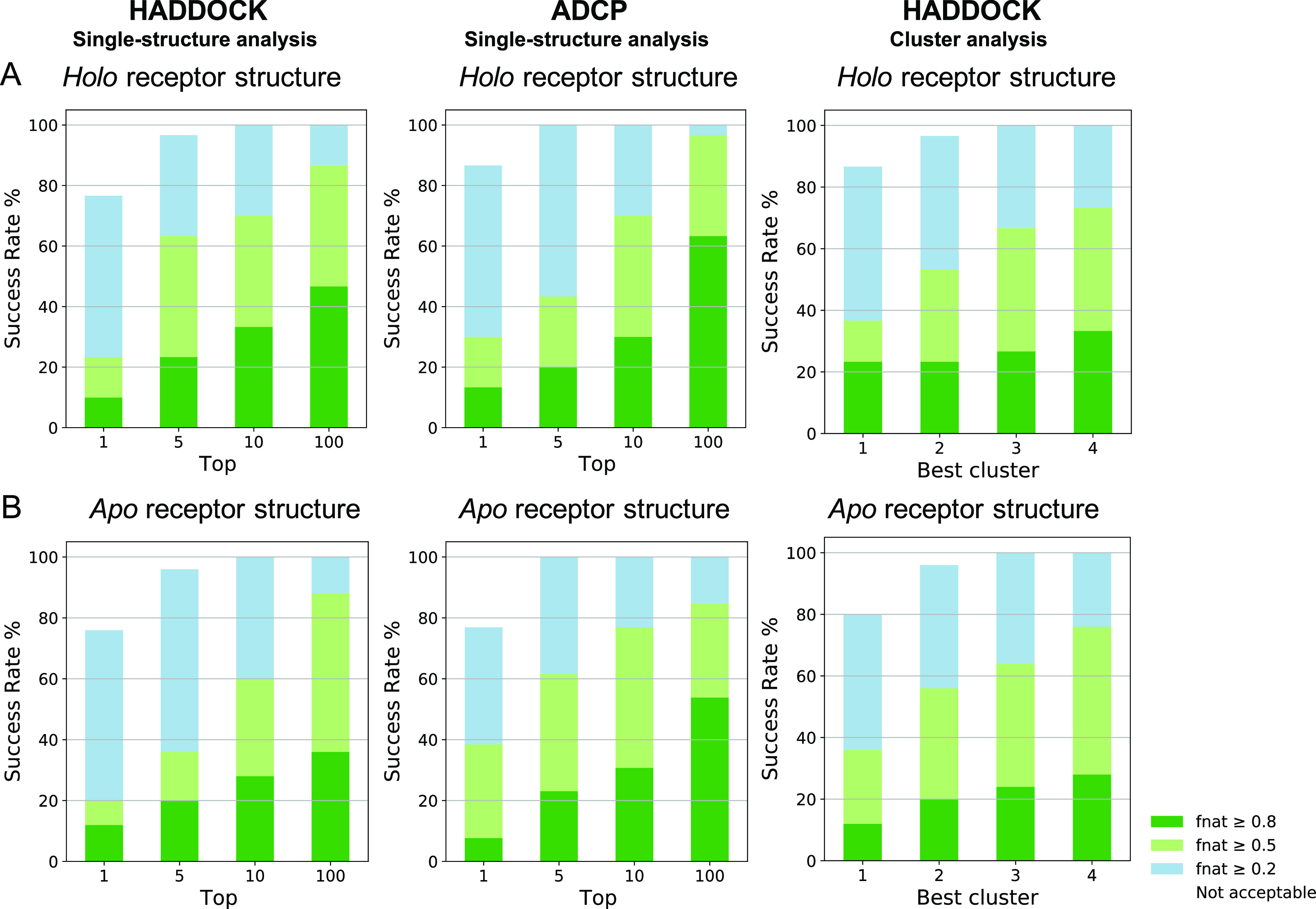
*F*_nat_ success-rate (%) plots to compare the performance of our optimized protocol with ADCP. Plotted are the success rates of ADCP and HADDOCK using either single-structure analysis or cluster analysis. In order to generate these graphs, the same dataset was used for both ADCP and HADDOCK. (A) Plotted are the success rates from the holo receptor docking (30 complexes) and (B) apo receptor runs (25 complexes). Color coding indicates model quality (from acceptable to high) according to the CAPRI criteria.

When the cluster analysis is performed for the HADDOCK results, the success rate of the best three clusters is already competitive with that of ADCP, even in the challenging unbound docking scenario (Figure 8). HADDOCK’s performance in the best three and best four clusters becomes 66.7 and 73.3%, respectively, in the holo condition and 64 and 76% for the apo setup (Figure 8).

**Figure 9:**
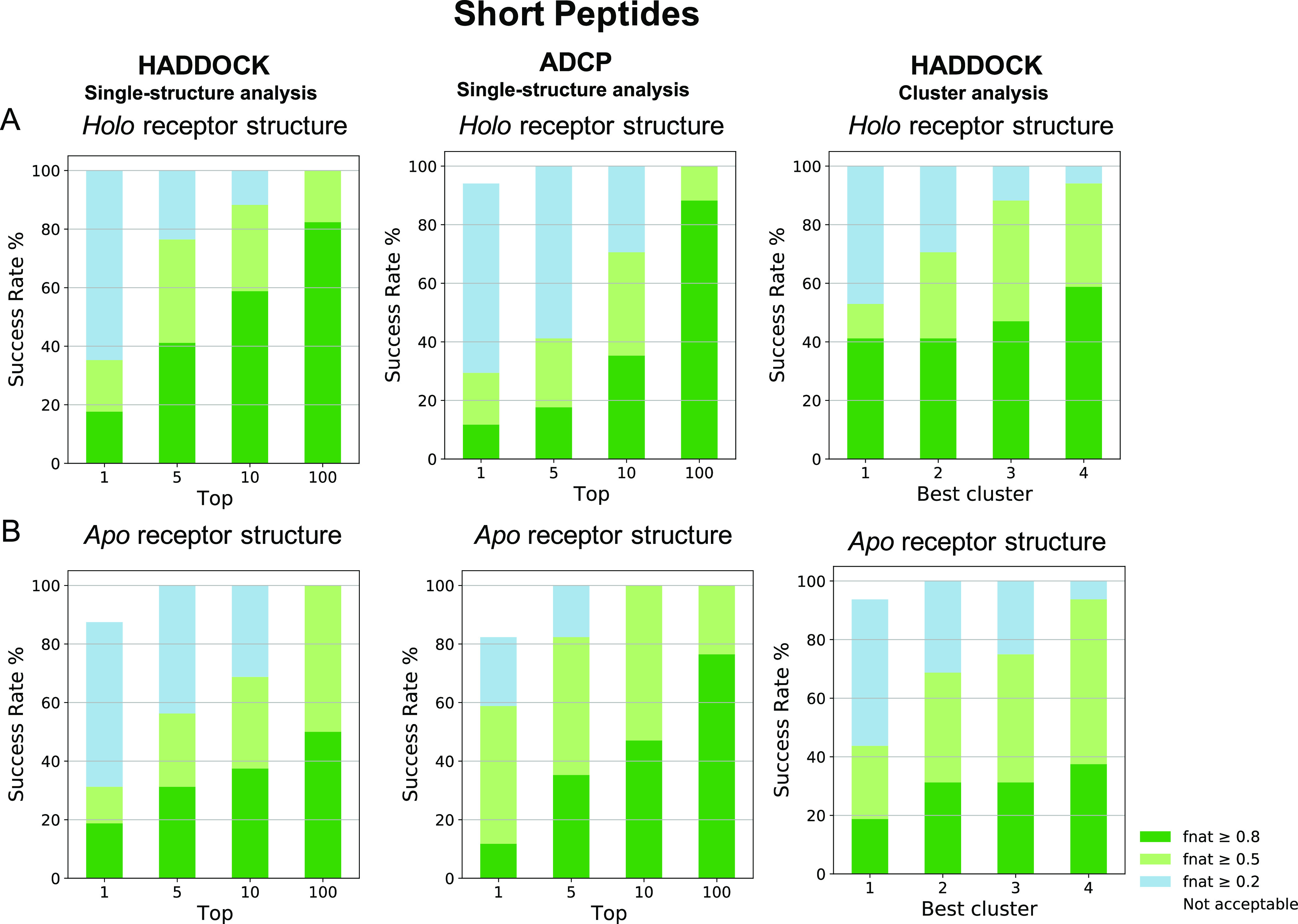
*F*_nat_ success-rate (%) plots for the short peptides in the two datasets. Plotted are the success rates of HADDOCK using the single-structure and cluster analysis and ADCP docking to the (A) holo (*n* = 17) or (B) apo receptor structure (*n* = 16). Color coding indicates model quality (from acceptable to high) according to the CAPRI criteria.

When only the short peptides are considered (17 in total), the prediction’s performance improves compared to the complete dataset, reaching a medium-to-high-quality success rate of 88.2 and 64.7% (top 10) for the holo and apo receptors, respectively. These scores are competitive with the ADCP results. The performance for acceptable or better models is also in line with ADCP and even slightly better (1 complex difference) for the top one (Figure 9). In the cluster analysis, the success rate of HADDOCK for medium- or high-quality structures within the best four clusters becomes 94.1% using the holo receptor and 93.8% using the apo, a performance that is similar (or better in the holo receptor case) to that of ADCP. Overall, our results show that HADDOCK’s cyclic peptide–protein docking performance is similar to that of ADCP even in the challenging fully unbound docking condition. Performance is also comparable when considering only the clinically relevant^48^ short peptides (≤10 residues), making HADDOCK a powerful tool for cyclic peptide–protein structure prediction.

## Conclusions

In this study, a step-by-step cyclization and docking protocol based on the integrative modeling software HADDOCK2.4 has been described. By starting from the sequence of the cyclic peptides, conformational ensembles were generated, which were subsequently used for docking together with the respective holo/apo forms of the receptor. Analysis of the various protocols shows that peptides of different lengths should be treated differently, mainly to address the conformational flexibility challenge posed by longer peptides (>10 residues).

The ability of HADDOCK to predict cyclic peptide–protein complexes is comparable to the state-of-the-art practice in the field based on ADCP. Most importantly, HADDOCK performs similarly to ADCP in the case of fully unbound docking and even slightly better when assessing the performance on a cluster basis (the default mode in HADDOCK). Since HADDOCK can also incorporate a variety of experimental data (e.g., NMR information about the peptide conformation) to guide the docking, the reported performance can be considered a lower limit, which can be enhanced by additional experimental input. In terms of computational efficiency, both ADCP and HADDOCK require a few hours or less to dock the peptides, depending on the number of CPUs available and the processor speed. As an indication, the computing time to generate one model for the three docking stages (it0/it1/itw) ranges from 8 to 47, 321 to 1898, and 5 to 874 s, respectively, on an AMD Opteron(tm) Processor 6320, depending on the receptor size and peptide length (longer peptides undergo the extended refinement protocol). For the peptide–protein docking with HADDOCK, users can also submit docking runs to the user-friendly web server (https://wenmr.science.uu.nl/haddock2.4), which makes use of the European Open Science Cloud high-throughput computing resources.^49^ Unfortunately, the generation of the cyclic peptide ensemble prior to docking is not yet supported by the web server and should be run locally for the time being.

Finally, it is worth noting that the presented HADDOCK protocol performs particularly well in predicting short cyclic peptide–protein complexes (≤10 residues), which constitute the majority of cyclic peptides in the clinic.^48^ Thus, the presented cyclization and docking protocol should be a valuable tool for the rational design of clinically relevant cyclic peptides.

## Supporting Information Available

The dataset used for this research and analysis scripts are freely available at https://github.com/haddocking/cyclic-peptides and the docking models are available from the SBGrid data reposity (https://data.sbgrid.org/dataset/912). The Supporting Information is available free of charge at https://pubs.acs.org/doi/10.1021/acs.jctc.2c00075.

Details of the peptide–protein complexes in the backbone and the disulfide dataset; parameters that have been changed in the run.cns and generate.inp files for step 2 and step 3 of cyclization, as well as for docking; description of the difference between an *f*_nat_ and an i-rmsd analysis for the cyclic peptide–protein complexes; rmsd analysis of the peptide conformational ensemble for the backbone and the disulfide dataset; success-rate plots of the 50STR_COMB protocol, comparing single-structure analysis and cluster analysis; success-rate plots of the 60STR protocol; success-rate plots of different docking protocols that all include a maximum of 50 peptide structures in the docking input; *F*_nat_ quality for each structure in the single-structure analysis for different docking protocols that all include a maximum of 50 peptide structures in the docking input; structural quality analysis of the generated models from the optimized docking experiment (50STR_COMB); success-rate plots of the optimized protocol (50STR_COMB) for the backbone and disulfide dataset in the holo receptor (bound) condition; and success-rate plots of the optimized protocol (50STR_COMB) in comparison to ADCP’s performance (PDF)
*f*_nat_ and i-rmsd values for the top 10 models (according to the HADDOCK itw score) for every complex of the dataset generated by the optimized protocol (50STR_COMB) (XLSX)
*f*_nat_ and i-rmsd values for the top 10 models (according to the HADDOCK itw score) for every complex of the dataset generated by the optimized protocol (50STR_COMB) (XLSX)
*f*_nat_ and i-rmsd values for the top 10 models (according to the HADDOCK itw score) for every complex of the dataset generated by the optimized protocol (50STR_COMB) (XLSX)
*f*_nat_ and i-rmsd values for the top 10 models (according to the HADDOCK itw score) for every complex of the dataset generated by the optimized protocol (50STR_COMB) (XLSX)
